# Carbapenem-resistant Enterobacterales susceptibility patterns to new antimicrobials: A single-center analysis

**DOI:** 10.1017/ash.2023.337

**Published:** 2023-09-29

**Authors:** Miranda Monk, Sarah Turbett, Christine Yang, Ramy Elshaboury

## Abstract

**Background:** Multidrug-resistant bacteria are of high concern, and empiric antimicrobial choice for infections caused by these pathogens, while awaiting susceptibilities, is increasingly encountered. We describe the susceptibility patterns of ceftazidime-avibactam (CZA), imipenem-rel-ebactam (I-R), meropenem-vaborbactam (MVB), cefiderocol (FDC), ceftolozone-tazobactam (C/T), minocycline (MIN), and tigecycline (TGC) for carbapenem-resistant Enterobacterales at an academic medical center. **Methods:** We performed a single-center analysis of Enterobacterales isolates from 110 hospitalized adult patients who had CZA, I-R, MVB, FDC, MIN, or TGC susceptibility testing performed between October 2020 and September 2022. The study included 1 isolate per patient per infection site per year. Isolates were divided into carbapenem susceptible and non susceptible categories. For carbapenem nonsusceptible isolates, phenotypic confirmatory testing of carbapenem nonsusceptibility was performed using disk diffusion, gradient diffusion, and/or broth microdilution. Interpretive categories were applied using CLSI- or FDA-approved break-points where applicable. Carbapenemase testing was also performed using the modified carbapenem inactivation method (mCIM) and, where applicable, this testing was confirmed at the Massachusetts State Public Health Laboratory using genotypic methods. **Results:** In total, 125 unique isolates were reviewed: 34 meropenem-susceptible and 91 meropenem-intermediate or resistant isolates. CZA, I-R, MVB, and FDC were active against all tested meropenem-susceptible isolates; however, 50% of tested isolates were susceptible to C/T. MIN and TGC, when tested, were active against 2 of 11 isolates (18%) and 14 of 16 isolates (86%), respectively. Of 91 meropenem-nonsusceptible isolates, most tested isolates were susceptible to MVB (59 of 72, 82%), followed by CZA (63 of 82, 77%), I-R (8 of 11, 73%), FDC (9 of 16, 56%), and C/T (1 of 12, 8%). TGC retained activity against 78 of 81 (96%) tested isolates. In contrast, MIN retained activity against 8 of 45 isolates (18%). Additionally, all (28 of 28, 100%) isolates that were nonsusceptible to at least 1 novel agent (CZA, I-R, MVB, FDC, or C/T) remained susceptible to TGC. State laboratory confirmatory testing was available for 75 isolates. Of 43 mCIM-positive isolates, all 28 KPC-producing isolates were susceptible to CZA, I-R, MVB, FDC and TGC. **Conclusions:** Among Enterobacterales, CZA, MVB, and I-R retained activity against most non-NDM CRE isolates in this local analysis, with comparable susceptibilities. TGC demonstrated excellent susceptibility for CRE and meropenem-susceptible isolates, offering an alternative for nonbloodstream infections. Choice of empiric agent with a newβ-lactam, β-lactam–β-lactamase inhibitors, or TGC appear to be reasonable empiric therapeutic options at our institution. CT and MIN warrant confirmatory testing prior to use due to low susceptibility rates among meropenem nonsusceptible isolates.

**Disclosures:** None

